# Sinonasal Lobular Capillary Hemangioma of the Nasal Cavity: A Rare Giant Vascular Tumor Managed With Preoperative Embolization and Medial Maxillectomy

**DOI:** 10.7759/cureus.93111

**Published:** 2025-09-24

**Authors:** Wajeeha Naveed, Salman Ahmed, Shamsa Hussain, Sahina Marium, Murtaza Ahsan Ansari

**Affiliations:** 1 Otolaryngology - Head and Neck Surgery, Civil Hospital Karachi, Dow University of Health Sciences, Karachi, PAK; 2 Otolaryngology - Head and Neck Surgery, Dow International Medical College, Dow University of Health Sciences, Karachi, PAK; 3 Radiology, Dow International Medical College, Dow University of Health Sciences, Karachi, PAK

**Keywords:** endoscopic excision, giant lobular capillary hemangioma, lobular capillary hemangioma, nasal mass, preoperative embolization, sinonasal tumor

## Abstract

Lobular capillary hemangioma (LCH), also known as pyogenic granuloma, is a benign vascular lesion most often found on the skin and oral mucosa. Its occurrence in the nasal cavity is uncommon, yet clinically significant because of its rapid growth, tendency to bleed, and similarity to more aggressive tumors. Although the exact cause is uncertain, trauma, hormonal influences, and vascular malformations have been suggested as contributing factors. We describe the case of a 41-year-old married woman with no known comorbidities who presented with progressive right-sided nasal obstruction, a gradually enlarging intranasal mass, recurrent episodes of epistaxis, and blood-stained thick nasal discharge for three months. On examination, a reddish, lobulated mass was seen filling the right nasal cavity and extending into the nasopharynx. The mass was tender and bled on touch. CT imaging showed a well-enhancing soft tissue lesion arising from the maxillary antrum with bony remodeling and erosion of the medial maxillary wall. Histopathology confirmed the diagnosis of LCH. The patient underwent medial maxillectomy via sublabial approach aided by endoscopic removal of the mass after preoperative embolization, which significantly reduced intraoperative bleeding. Recovery was uneventful. Though rare, sinonasal LCH should be considered when evaluating vascular nasal masses. Early diagnosis and complete excision remain the mainstay of management. In large or highly vascular lesions, preoperative embolization can be a valuable adjunct to improve surgical safety.

## Introduction

Lobular capillary hemangioma (LCH), commonly known as pyogenic granuloma, is a benign vascular lesion of unknown etiology. Although it most often affects the skin and oral mucosa, it rarely involves the nasal cavity and accounts for about 10% of all cases in the head and neck [1]. In the sinonasal tract, the LCH presents as a bleeding polypoidal mass, measuring around 0.9 cm on average in the largest diameter [2]. Patients often present with progressive nasal obstruction and recurrent episodes of epistaxis, clinical features that make it indistinguishable from more aggressive tumors, such as angiofibroma, inverted papilloma, and hemangiopericytoma. This necessitates prompt histopathological diagnosis and management. Furthermore, the vascular nature of this tumor increases the chances of bleeding during biopsy or intraoperatively. For this reason, preoperative angioembolization is advocated in cases with large and highly vascular lesions. We report a case of sinonasal LCH that was managed successfully by medial maxillectomy via the sublabial approach, assisted with endoscopic surgical excision following preoperative angioembolization. Our case highlights the role of preoperative embolization of vascular tumors in significantly reducing intraoperative bleeding during surgical excision, which has proven to be a valuable adjunct in the management of highly vascular sinonasal tumors [3].

## Case presentation

A 41-year-old married female with no known comorbidities presented to the ENT department of Dow University Hospital, Karachi, Pakistan, with complaints of progressive unilateral nasal obstruction, recurrent episodes of epistaxis, and blood-stained, foul-smelling nasal discharge for three months. According to the patient’s attendant, she had been in her usual state of health until a small intranasal swelling was noticed, which gradually increased to the size of a small lemon. It was associated with facial pain, tenderness, and decreased sensation of smell.

There was no history of trauma, anosmia, fever, postnasal drip, weight loss, radiation exposure, or systemic illness. Past surgical history was significant for abdominal surgery 20 years ago. There was no family history of similar conditions. Personal history revealed decreased appetite and constipation.

Examination: The patient’s entire face was tender. Nasal airflow was absent on the right side, and there was marked tenderness over the right frontal and maxillary regions and the bridge of the nose. Anterior rhinoscopy revealed a reddish, lobulated, firm mass occupying the right nasal cavity and extending posteriorly into the nasopharynx. The mass was non-reducible and bled upon touch. No cervical lymphadenopathy was detected. Oral cavity examination was clear.

**Figure 1:**
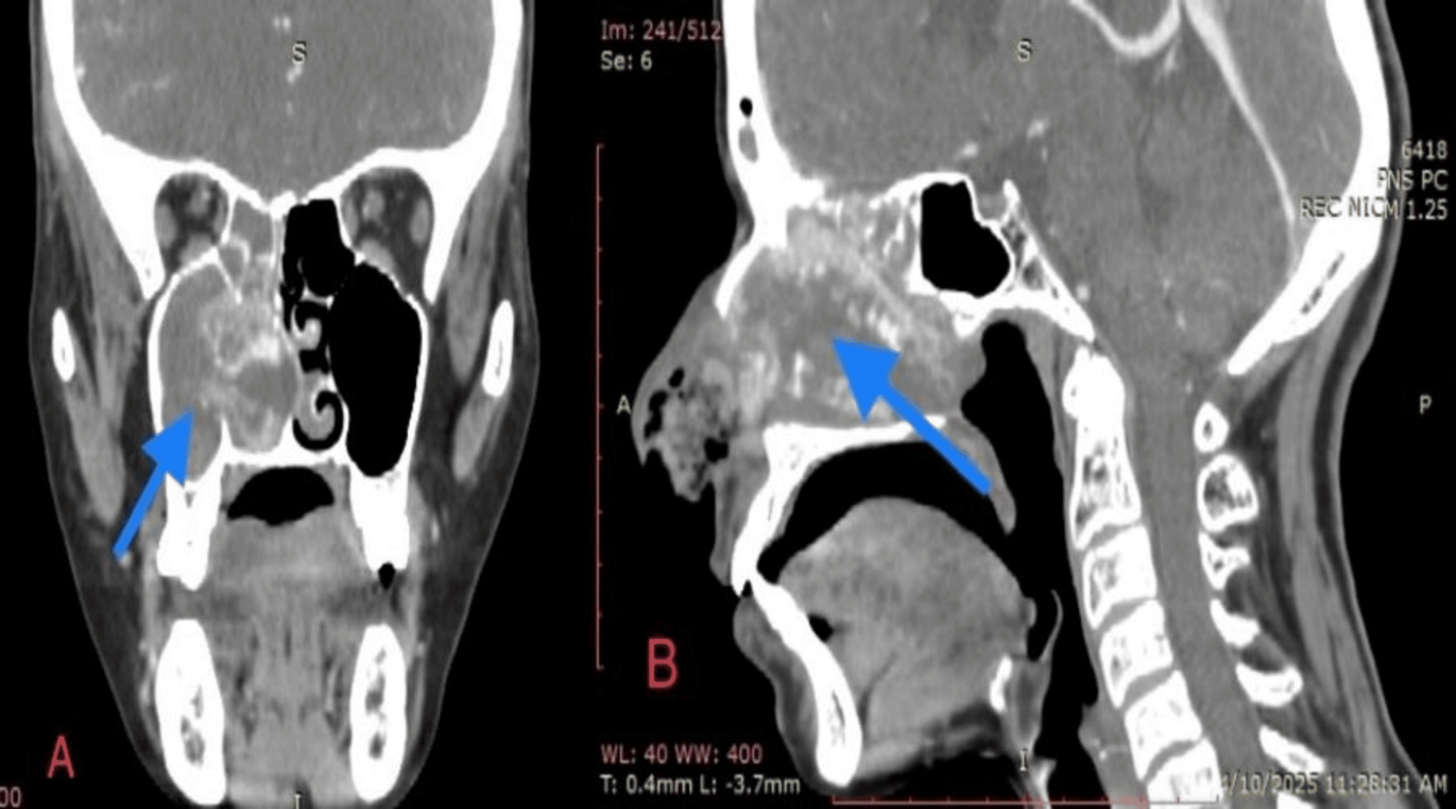
Coronal (A) and sagittal (B) planes of a contrast-enhanced CT scan of the nose and paranasal sinuses A contrast-enhanced CT scan of the nose and paranasal sinuses revealed enhancing polypoidal mucosal thickening and total tissue opacification of the right frontal, ethmoidal, maxillary, and sphenoid sinuses, with the epicenter at the right maxillary antrum. It was associated with widening of the osteomeatal complex and appeared to merge with the right nasal turbinates, almost totally obliterating the right nasal cavity. This resulted in remodeling and expansion of the sinonasal cavity, producing contralateral mass effect and extending up to the posterior choanae. Multifocal rarification and erosion of the medial wall of the maxillary sinus with erosion of the intervening bony septa were noted. No definite intraorbital or intracranial extension was seen.

Investigations: A CT scan of the nose and paranasal sinuses showed an extensive, enhancing soft tissue density mass in the right nasal cavity, extending into the maxillary antrum, posterior choana, and nasopharynx, with bony remodeling, expansion of the sinonasal cavity, rarification, and erosion of the medial wall of the maxillary sinus (Figure 1).

**Figure 2:**
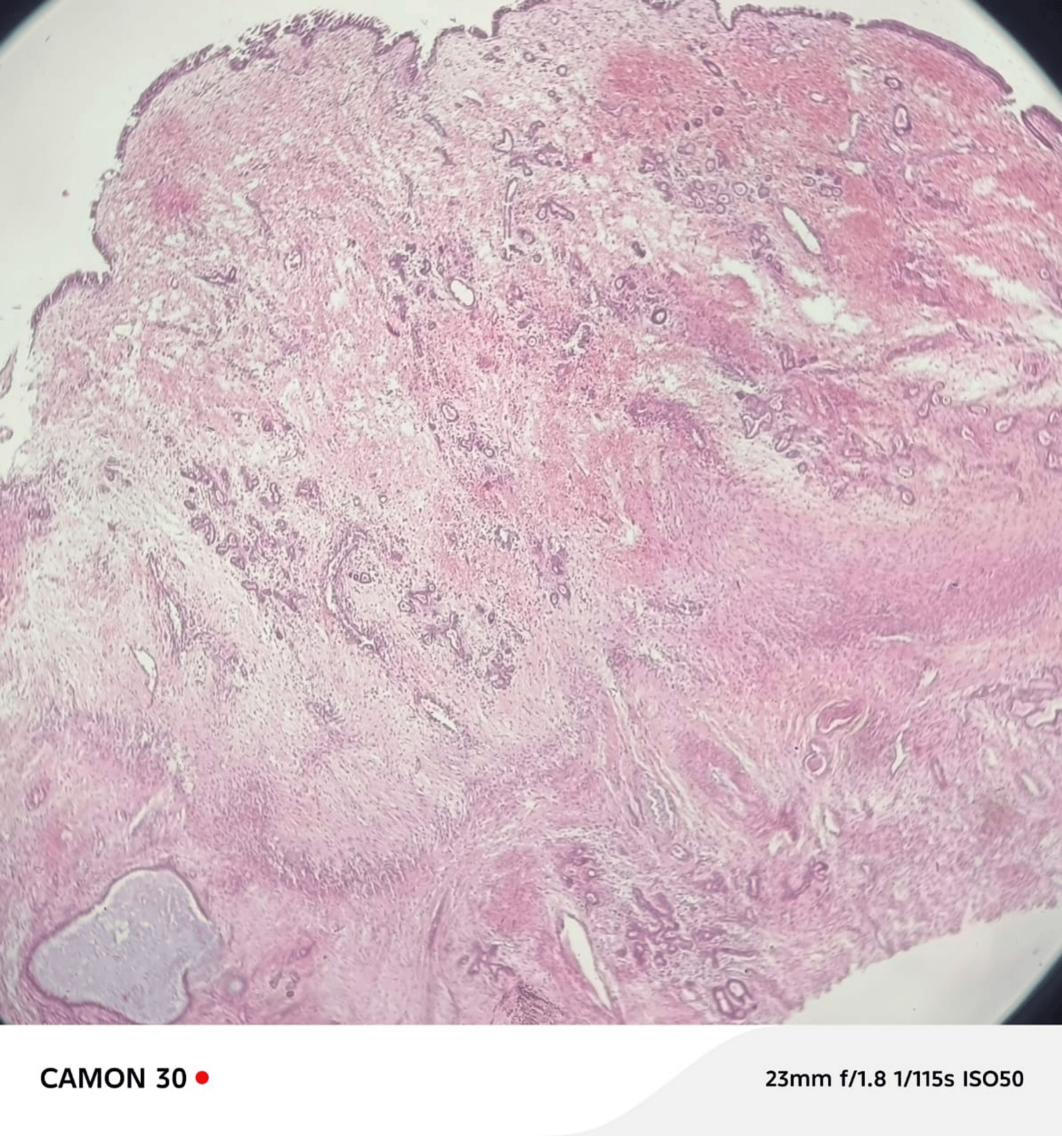
Histopathology image of the tumor showing vascular proliferation of capillary-sized vessels arranged in lobules, separated by the spindloid stroma covered with respiratory epithelium

Microscopic examination revealed lobular architecture composed of capillary-sized vessels arranged in lobules and separated by spindloid stroma. These findings were consistent with sinonasal LCH (Figure 2).

**Figure 3:**
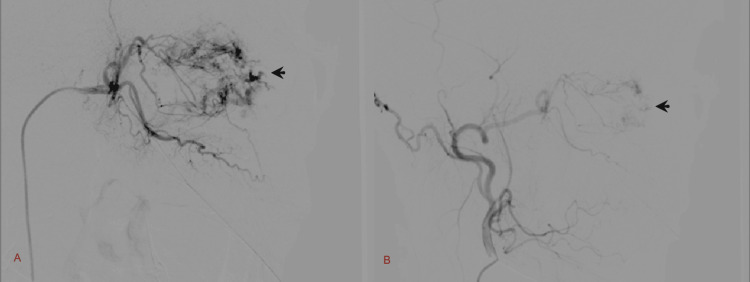
Spot images taken from preoperative embolization of sinonasal LCH Lateral view: (A) tumor blush seen with major supply from the branches of the right external carotid artery and minimal supply from the branches of the right anterior cerebral artery. (B) The same patient after selective angioembolisation of the right external carotid artery

Treatment: A carotid angiogram was done, which showed the tumor blush with major supply from branches of the right external carotid artery and minimal supply from the branches of the right anterior cerebral artery. Minimal supply of the tumor blush was also noted from the branches of the left external carotid artery. The branches of the right external carotid artery were selectively cannulated, and angioembolization was done using gel foam. The branches of the left external carotid were difficult to reach. Follow-up angiogram showed optimum embolization of the tumor vessels. The patient underwent preoperative angioembolization of the branches of the right external carotid artery to minimize the risk of intraoperative bleeding (Figure 3).

**Figure 4:**
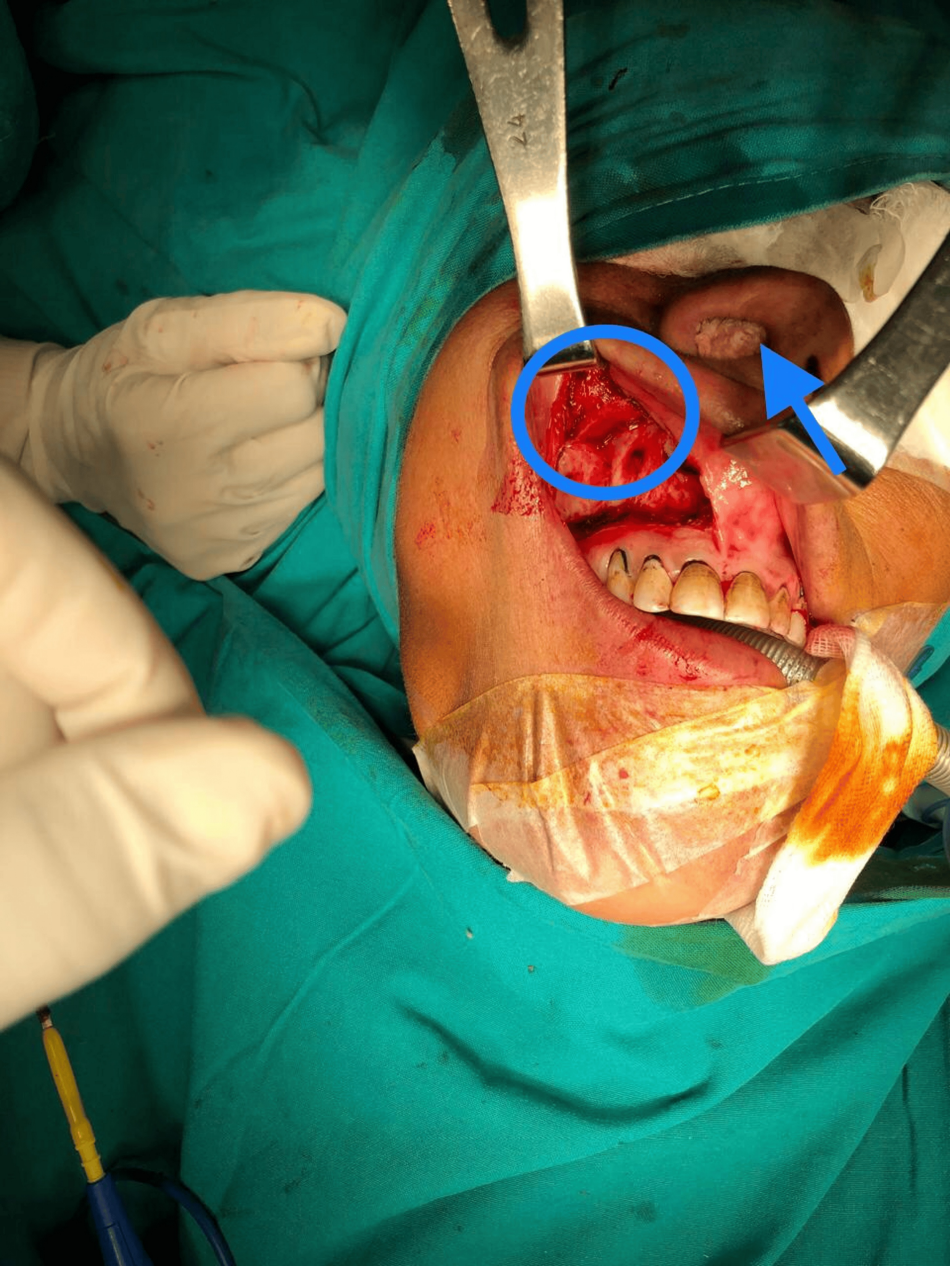
Intraoperative image showing the lobular capillary hemangioma (circle) being exposed during medial maxillectomy The lesion occupies the right nasal cavity (arrow) and is seen abutting the medial maxillary wall

**Figure 5:**
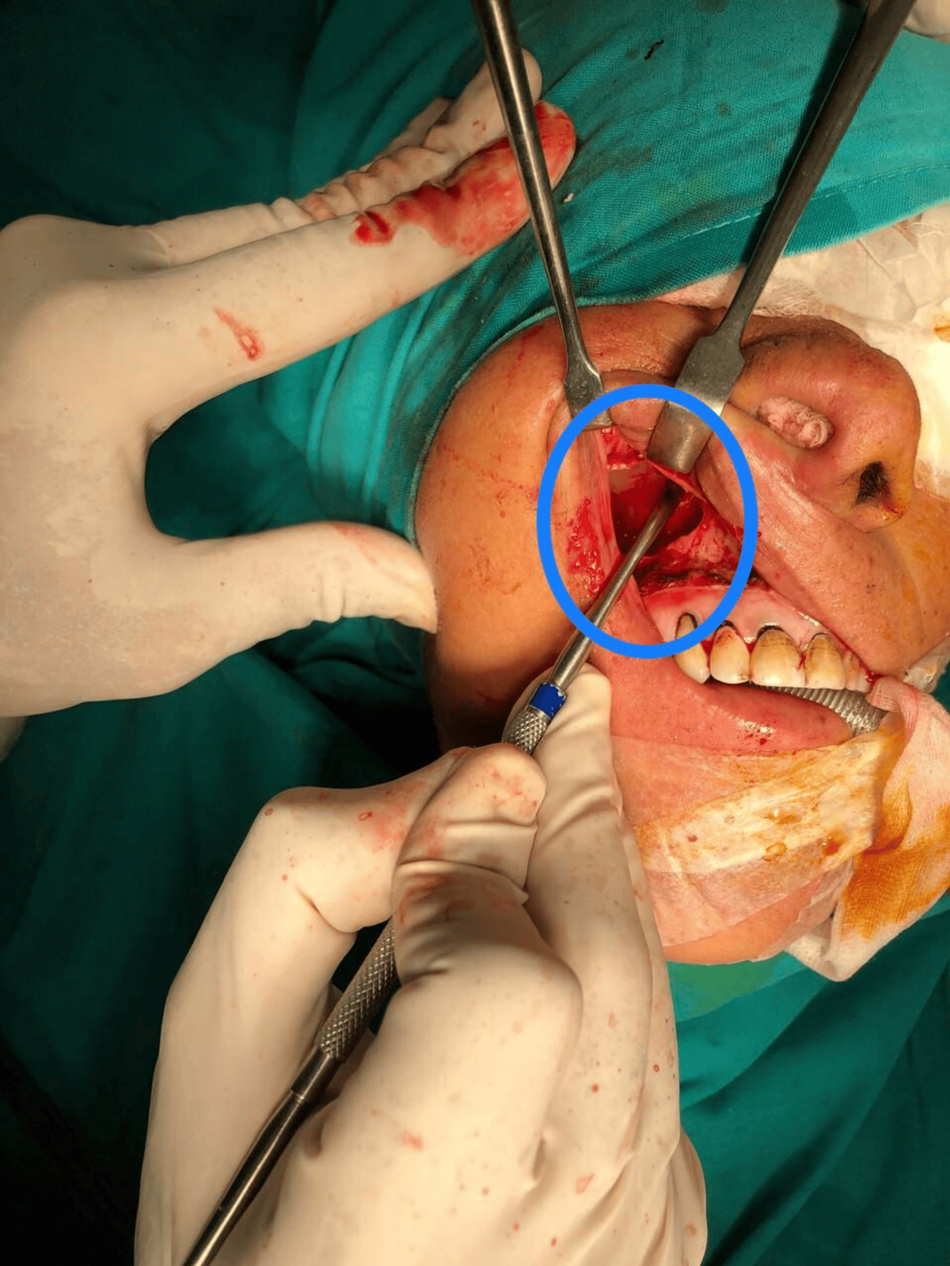
Transoral excision of the highly vascular sinonasal LCH through medial maxillectomy Surgical instruments are seen dissecting part of the tumor away from the maxillary sinus

The patient subsequently underwent complete excision of the mass through a medial maxillectomy performed via a sublabial incision under general anesthesia. A horizontal incision was placed in the gingivobuccal sulcus, and the mucoperiosteal flap was elevated to expose the anterior wall of the maxilla. Osteotomies were performed to remove the medial wall of the maxilla and provide adequate exposure to the lateral nasal wall. The mass was identified as highly vascular and attached to the lateral nasal wall. Careful dissection was carried out with coagulation of feeding vessels, and the lesion was excised in its entirety under endoscopic guidance. Meticulous hemostasis was achieved, and the sublabial incision was closed in layers (Figures 4-5).

Outcome: Postoperative recovery was uneventful. The patient was discharged in stable condition and scheduled for regular follow-up with endoscopic examinations due to the potential risk of recurrence. Follow-up at six months showed no recurrence.

## Discussion

LCH, also known as nasal pyogenic granuloma, is a rare benign vascular lesion. Although it frequently affects the oral mucosa and skin, the incidence of sinonasal LCH is reported to be less than ten percent of all cases [1]. The most common sites of origin in the nasal cavity are the anterior nasal septum, the turbinates, and the roof of the nasal cavity, in decreasing order. It is characterized by rapid growth, recurrent episodes of epistaxis, and marked friability. Clinically, affected patients often present with progressive nasal obstruction and recurrent bleeding, which may mimic more aggressive tumors. Histologically, it comprises lobules of capillary-sized vessels separated by fibrous septa, a feature that distinguishes it from other vascular masses of the head and neck. The exact pathophysiology remains unclear. However, nasal trauma, chronic irritation, hormonal influences, pregnancy, and angiogenic factors have all been suggested as possible contributing factors [4,5].

As this rare and benign vascular lesion often resembles more aggressive sinonasal masses clinically and radiologically, histopathological confirmation is crucial to differentiate between juvenile nasopharyngeal angiofibroma (JNA), hemangiopericytoma, and inverted papilloma. JNA typically affects adolescent males, arises from the sphenopalatine foramen, and is frequently associated with bony remodeling on imaging. Hemangiopericytoma also presents as a vascular nasal mass but can be distinguished histopathologically by its characteristic staghorn vascular pattern and pericytic proliferation, in contrast to the lobular architecture seen in LCH [6]. Inverted papilloma is another uncommon benign sinonasal tumor that usually occurs in middle-aged men, presenting with unilateral nasal obstruction, irregular polypoidal masses, epistaxis, and sinonasal pain, often with bony changes; histology demonstrates epithelial invagination rather than vascular lobules. Rare malignant vascular tumors such as Kaposi’s sarcoma and angiosarcoma must also be considered, as they exhibit atypia and infiltrative growth not seen in LCH. Other benign conditions, including antrochoanal polyps and organizing hematomas, may mimic LCH clinically; however, histopathological assessment remains definitive.

Several surgical approaches have been described for the management of sinonasal LCH. Historically, external procedures such as lateral rhinotomy, the Caldwell-Luc operation, and midfacial degloving provided good exposure for large or posterior lesions but were associated with significant morbidity, visible scarring, prolonged recovery times, and higher complication rates. With the development of modern endoscopic techniques, these external approaches are now rarely indicated [7,8].

Endoscopic endonasal excision has emerged as the preferred treatment for most cases of sinonasal LCH. It provides excellent visualization of both the lesion and its point of origin, allowing precise dissection with minimal trauma to adjacent structures. Endoscopy also enables concurrent cauterization of the vascular pedicle, thereby reducing intraoperative bleeding and lowering recurrence risk. Patients benefit from reduced morbidity, shorter hospital stays, and quicker recovery. Multiple published studies have confirmed high rates of complete excision and low recurrence with this technique, establishing it as the current gold standard [9,10].

However, in large, laterally based, or highly vascular lesions, purely endoscopic removal may be technically difficult or associated with a higher risk of incomplete excision or uncontrolled bleeding. In such cases, external approaches remain valuable.

**Figure 6:**
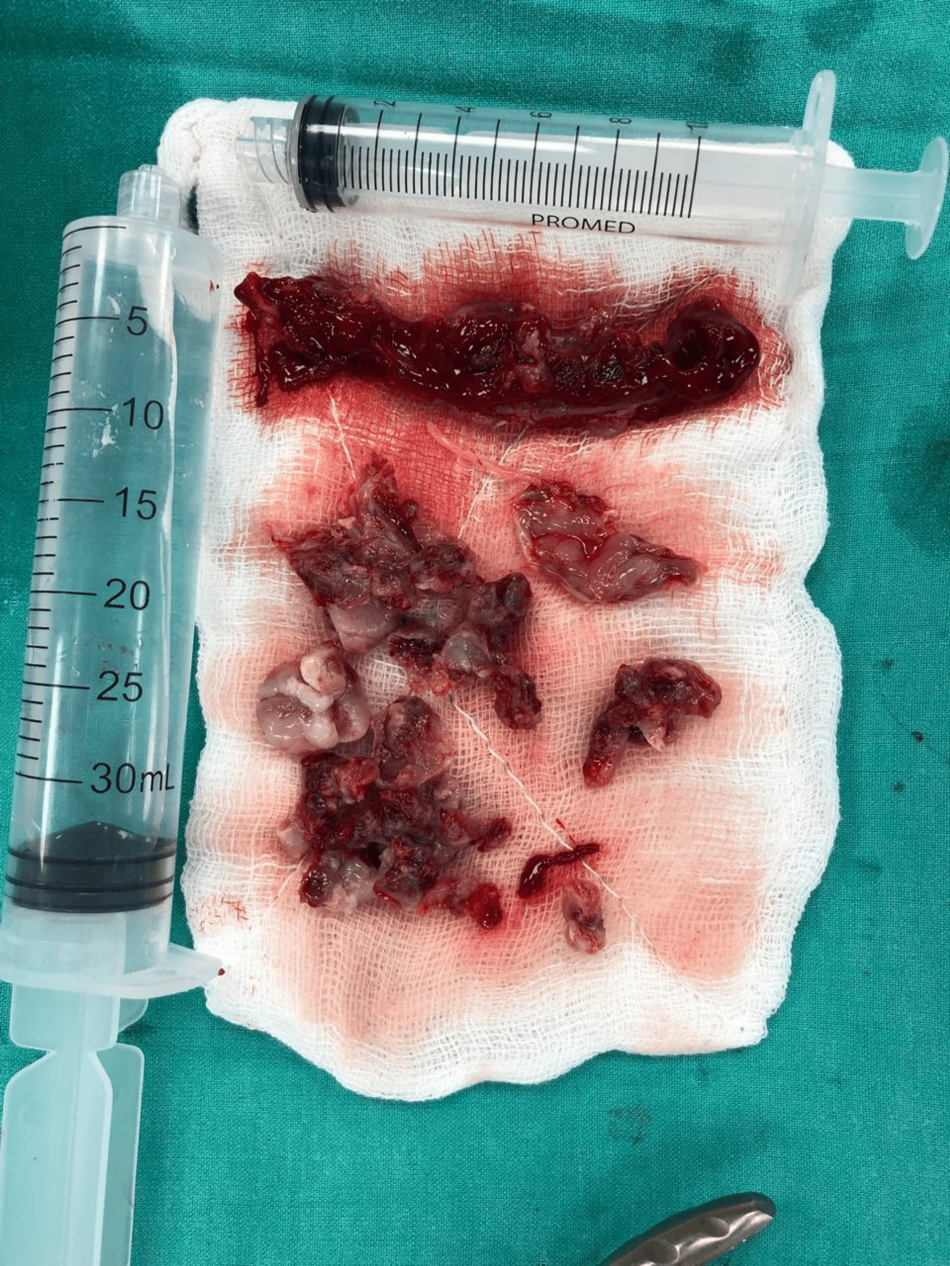
Excised specimen (gross morphology)

Surgical approaches also differ based on the tumor size and extent. Most sinonasal LCH tumours measure approximately 1-4.5 cm, as described by Alghamdi et al. and Lee et al. However, some sinonasal tumors that exceed 5 cm are classified as “giant” lesions, as reported by Choi et al. [13], and in other studies describing excised masses measured up to 10 cm [11-15] (Figure 6).

In our case, the excised sinonasal LCH measured approximately 6 × 3 cm in aggregate, which was classified as a giant lesion. For this reason, preoperative embolization followed by medial maxillectomy through a sublabial incision, aided by endoscopic removal, was performed, reducing intraoperative bleeding, allowing greater tumor exposure, and ensuring complete surgical resection. The combined method allowed wide exposure for safe mobilization and resection while still benefiting from the precision of endoscopic visualization. Preoperative embolization has been shown to significantly improve surgical safety in selected highly vascular sinonasal tumors [3,16]. However, endoscopic excision remains the most widely adopted treatment for small to moderate-sized sinonasal LCH, while external procedures such as lateral rhinotomy and medial maxillectomy are generally reserved for larger lesions or those with inaccessible extensions.

Because of the high vascularity of these lesions, intraoperative bleeding remains a major concern. Although we employed preoperative embolization of the mass, other adjunctive strategies, including intraoperative cautery and hypotensive anesthesia, have also been reported. Vascular and interventional radiology (VIR)-guided embolization is particularly useful for large, highly vascular lesions where excessive hemorrhage is anticipated. While embolization is not necessary in every case, its selective use in extensive or vascular sinonasal hemangiomas is a valuable adjunct to surgery [15,16].

The prognosis of sinonasal LCH is excellent following complete surgical excision. The rate of recurrence depends on the follow-up duration and the case series, ranging between 0% and 42%. Recurrences are typically related to incomplete resection or persistent predisposing factors such as trauma or hormonal influences. Regular endoscopic follow-up is strongly recommended to allow early detection of recurrence [9].

## Conclusions

This case highlights the importance of considering sinonasal LCH in the differential diagnosis of nasal masses presenting with recurrent epistaxis and progressive obstruction. Prompt diagnosis, selective use of adjuncts such as preoperative embolization, and complete surgical excision - whether endoscopic or through external approaches such as medial maxillectomy - lead to excellent outcomes with minimal recurrence.

Furthermore, our case describes a giant sinonasal LCH managed with preoperative embolization followed by medial maxillectomy via the sublabial approach and endoscopic removal. To the best of our knowledge, only a few reports have utilized this approach in giant LCH of the sinonasal cavity, demonstrating its safety and effectiveness.
